# DLA-Net: dual lesion attention network for classification of pneumoconiosis using chest X-ray images

**DOI:** 10.1038/s41598-024-61024-3

**Published:** 2024-05-21

**Authors:** Md. Shariful Alam, Dadong Wang, Arcot Sowmya

**Affiliations:** 1 School of Computer Science and Engineering, University of New South Wales, Sydney, Australia; 2 CSIRO Data61, Sydney, Australia

**Keywords:** Cardiovascular diseases, Health care, Health occupations, Medical research

## Abstract

Accurate and early detection of pneumoconiosis using chest X-rays (CXR) is important for preventing the progression of this incurable disease. It is also a challenging task due to large variations in appearance, size and location of lesions in the lung regions as well as inter-class similarity and intra-class variance. Compared to traditional methods, Convolutional Neural Networks-based methods have shown improved results; however, these methods are still not applicable in clinical practice due to limited performance. In some cases, limited computing resources make it impractical to develop a model using whole CXR images. To address this problem, the lung fields are divided into six zones, each zone is classified separately and the zone classification results are then aggregated into an image classification score, based on state-of-the-art. In this study, we propose a dual lesion attention network (DLA-Net) for the classification of pneumoconiosis that can extract features from affected regions in a lung. This network consists of two main components: feature extraction and feature refinement. Feature extraction uses the pre-trained Xception model as the backbone to extract semantic information. To emphasise the lesion regions and improve the feature representation capability, the feature refinement component uses a DLA module that consists of two sub modules: channel attention (CA) and spatial attention (SA). The CA module focuses on the most important channels in the feature maps extracted by the backbone model, and the SA module highlights the spatial details of the affected regions. Thus, both attention modules combine to extract discriminative and rich contextual features to improve classification performance on pneumoconiosis. Experimental results show that the proposed DLA-Net outperforms state-of-the-art methods for pneumoconiosis classification.

## Introduction

Pneumoconiosis is an occupational lung disease caused by excessive exposure to respirable particles such as coal, silica and asbestos, and the disease caused by the inhalation of coal dust is also known as black lung or Coal Workers’ Pneumoconiosis (CWP)^1^. In 2013 alone, globally approximately 260,000 people died due to this disease^2^. The lung transplantation rate is also increasing among pneumoconiosis patients^3^. Moreover, recent reports indicate that dental technicians are also affected by pneumoconiosis due to the inhalation of different airborne particles^4^. Pneumoconiosis is not curable but preventable; therefore, regular screening of workers at potential risk is crucial for monitoring, early intervention and prevention.

Current clinical diagnosis of pneumoconiosis is mainly based on chest X-ray (CXR) images due to the low-dose radiation that reduces cross infection risk in the radiology department, relatively low cost and wide availability^5^. According to the International Labour Organization (ILO) guidelines^6^, pneumoconiosis can be grouped into four main categories, namely cat-0, cat-1, cat-2 and cat-3, based on the profusion of small opacities observed in the lung regions. Cat-0 refers to the absence of small opacities or the presence of small opacities that are less profuse than cat-1, while cat-3 refers to the most significant levels of profusion. ILO guidelines greatly facilitate the diagnosis of pneumoconiosis; however, manual diagnosis using CXR images requires a large number of well trained and experienced radiologists, which is expensive. In addition, manual diagnosis is laborious and time-consuming as radiologists need to interpret the subtle appearance of opacities on CXR images, and is prone to human errors due to low contrast of the CXR image and visual similarity between different classes^7^. Therefore, developing a reliable Computer-Aided Diagnosis system (CAD) for pneumoconiosis is critical for accurate and fast detection at a relatively low cost.

**Figure 1:**
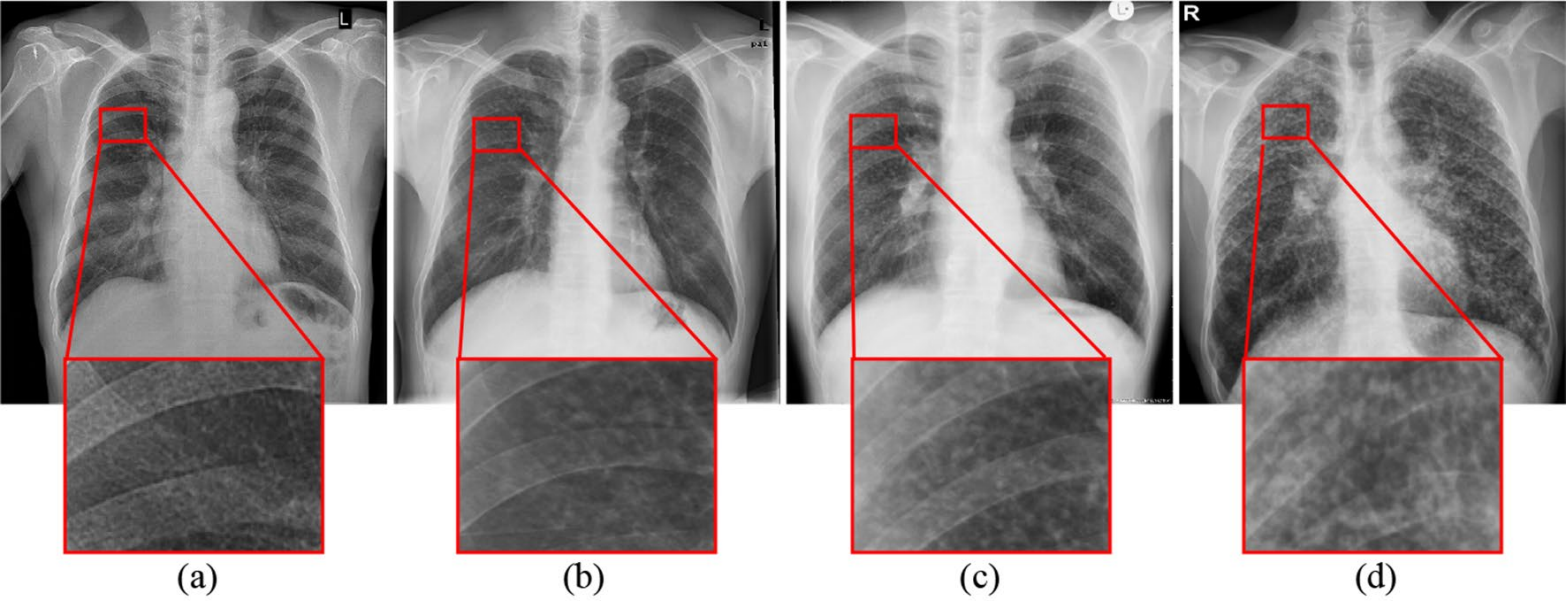
Examples of four different categories of pneumoconiosis based on the profusion of small opacity lesions. (**a**) cat-0, (**b**) cat-1, (**c**) cat-2, and (**d**) cat-3. Red bounding boxes correspond to densities of small opacities in images of different categories.

**Figure 2:**
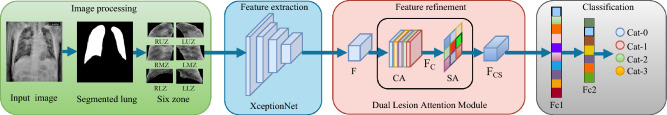
Proposed DLA-Net architecture with a dual lesion attention (DLA) module based on an ImageNet-pretrained Xception Net^20^ architecture, consists of four main components: image processing that segments the lung field into six zones, feature extraction that uses ImageNet-pretrained Xception Net^20^ to extract semantic information, feature refinement that consists of a dual lesion attention (DLA) module to focus on lesion regions, and classification that classifies pneumoconiosis into one of four ILO categories using a fully connected (FC) layer. CA: channel attention, SA: spatial attention.

Developing a radiograph-based CAD systems is very challenging because of: (1) low contrast and visual similarities of opacities that lead to inter- and intra-observer variations^5,7^ (as shown in Fig. 1), (2) the presence of artifacts in the CXR images, (3) overlap of other diseases, and (4) small and relatively imbalanced dataset due to disease occurrence and limitation on data sharing. In the last few decades, researchers have devoted considerable effort to develop radiograph-based CAD systems^5,8,9^. Most existing CAD systems for pneumoconiosis are machine learning-based that depend on handcrafted features such as Fourier spectrum^10^, texture descriptors^11^, histogram analysis^12^, k-Nearest Neighbours (kNN)^13^, Support Vector Machines (SVM)^14^ and Artificial Neural Networks (ANN)^10^. For example, Richard et al.^15^ developed a texture analysis-based method for the identification of pneumoconiosis from posterior anterior X-ray images. Katsuragawa et al.^16^ applied the Fourier transform for the diagnosis of CWP using CXR images. An amplitude-modulation frequency-modulation (AM-FM) based CAD was proposed by Murray et al.^12^ for the detection of pneumoconiosis using radiographs. Yu et al.^17^ also presented a histogram analysis-based pneumoconiosis detection system. Okumura et al.^10^ exploited power spectra to classify pneumoconiosis using ANN. They developed pattern recognition plus rule-based techniques to classify pneumoconiosis using radiographs. However, these machine learning-based methods are limited in capturing powerful discriminative features to address the challenging task of pneumoconiosis classification^5^.

Due to the automatic feature extraction capabilities and end-to-end training strategy, deep learning methods such as Convolutional Neural Networks (CNN)-based approaches have replaced the dominant position of the traditional machine learning algorithms for pneumoconiosis classification^5,8,9^. In recent years, some works have investigated CNN-based methods that outperform the traditional machine learning-based methods for pneumoconiosis classification^5^. Zhang et al.^8^ developed a CNN-based model to detect and classify pneumoconiosis into four different stages using CXR images. They first segmented the lung field into six zones. Then the ResNet-101 model was used to predict the opacity level for each zone separately. Finally, the classification of each subject was determined by summarising the prediction results of the six zones. In the same year, Yang et al.^9^ proposed a two-stage pipeline to classify pneumoconiosis. First, they segmented the lung field in CXR images using U-Net and then used ResNet-34 to extract features from the lung regions for classification. In another study, Devnath et al.^18^ compared the performance of seven CNNs including InceptionV3^19^, Xception^20^, ResNet50^21^ and DenseNet121^22^ to classify pneumoconiosis. All these studies demonstrate the effectiveness and feasibility of deep learning-based models in the diagnosis of pneumoconiosis.

Although CNN-based methods have shown improved performance, severity analysis of pneumoconiosis using CXR images still suffers in the area of feature extraction from infected regions with complex shapes, and this limited performance hinders real-world applications of these systems^5^. As shown in Fig. 1, there is some similarity in terms of geographical features between the three categories of pneumoconiosis. In addition, infected regions occupy only a small part of the whole image, and are usually surrounded by non-lesion regions. Existing CNN-based methods cannot focus on distinguishing critical information that is crucial to multi-class classification of pneumoconiosis. Therefore, the main challenge of pneumoconiosis classification is the extraction of discriminative features from infected lung regions.

To address these challenges, we propose a CNN-based pneumoconiosis classification network that uses dual lesion attention modules, denoted as the Dual Lesion Attention Network (DLA-Net), which employs two attention modules, called the channel attention (CA) and spatial attention (SA) modules, to improve discriminative feature extraction capability by paying attention to lesion areas inside the lung regions in CXR images. Recently, the attention mechanism has been successfully utilised in medical image related tasks, e.g., classification^23^, and segmentation^24^. The main idea behind the attention mechanism is to focus on the most salient parts of the features in images and suppress irrelevant information. Hu et al.^25^ proposed a channel-wise attention mechanism in the squeeze and excitation (SE) block and achieved promising performance. The channel attention mechanism uses global average-pooling to recalibrate the inter-channel dependencies on different channels of the feature maps. Woo et al.^26^ combined both channel and spatial attention maps in a Convolutional Block Attention Module (CBAM) to recalibrate the intermediate features. Since then, channel-wise and spatial-wise attention mechanisms have been used to recalibrate fully convolutional networks. The superior discrimination capability of attention mechanisms motivate the integration of attention modules with a CNN backbone to further improve feature extraction capabilities. In the proposed DLA-Net, Xception^20^ pre-trained on the ImageNet dataset is used as a backbone to extract features. The backbone features are refined using two attention modules, including the channel attention (CA) module to improve representation quality by recalibrating the inter-channel dependencies between the channels of its feature maps, and the spatial attention (SA) module to highlight the salient location in the feature maps. The performance of other backbone feature extraction models integrated with the DLA module was also investigated, and Xception Net^20^ was found to achieve better performance than other backbone models (See Table 5).

*Contributions* The main contributions of this work are summarised as follows:

1. DLA-Net is proposed for classification of pneumoconiosis based on the profusion of small opacities and state-of-the-art results were achieved for pneumoconiosis classification.
2. The proposed architecture includes channel attention and spatial attention modules to focus on the most important channels in the feature maps and highlight the spatial details of the infected regions in chest X-rays.
3. Extensive ablation studies have been performed to verify the effectiveness of the proposed DLA-Net for pneumoconiosis classification.

## Methodology

As discussed in the previous section, pre-trained CNN models have achieved significantly improved performance compared to the traditional machine learning-based methods on pneumoconiosis classification^5^. However, these architectures often fail to extract powerful discriminative features and therefore, still exhibit unsatisfactory classification performance. To address this problem, the DLA module is employed which incorporates both the CA module to prioritise important ‘what’ information and the SA module to pinpoint the location of the crucial information. The DLA module was integrated into Xception Net to extract discriminative information. The architecture of the proposed zone-based classifier is depicted in Fig. 2. The framework consists of four components: image processing, feature extraction, feature refinement and classification. In the following subsections, these components are described.

*Image processing* The scarcity of computing resources at pneumoconiosis screening sites renders impractical the development and application of deep learning models that work on whole high-resolution CXR images. There are two possible solutions to consider: either decreasing the model capacity (e.g., by utilising shallow learning techniques) or down-sampling the images. Since pneumoconiosis diagnosis relies heavily on subtle image features, compromising the richness of these features through model simplification or reducing image resolution is not ideal. Instead, by adopting the method proposed by Zhang et al.^8^, the lung fields are divided into six zones. Each zone is classified individually, and subsequently the classification results of the zones are combined to derive an overall image classification score. Six individual models for six zones were trained separately to classify each zone of an X-ray image into an ILO category.

The image processing component is shown leftmost in Fig. 2. First, the lung fields were segmented from the CXR images using the DCI-UNet model^27^. To segment the lungs, the downsized images were fed to the model, and the resulting masked images were upsized to the original image size. After segmentation, each lung field was divided into three zones by dividing the vertical distance between the lung apex and the dome of the diaphragm into three equal parts and drawing a horizontal line at each division point. For easy reference, the algorithm assigns each zone a label, which are Right Upper Zone (RUZ), Right Middle Zone (RMZ), Right Lower Zone (RLZ), Left Upper Zone (LUZ), Left Middle Zone (LMZ) and Left Lower Zone (LLZ). Six zone classifiers were trained separately to classify each zone of the X-ray image into an ILO category.

*Feature extraction* DLA-Net was evaluated using Xception^20^ as the backbone network pre-trained on ImageNet dataset^28^. The models were initialised with pre-trained weights and then finetuned using the training data. Alternative CNNs were also investigated as backbone networks. However, it was found that Xception Net^28^ outperformed other backbone networks, yielding superior results (See Table 5). From the backbone model, feature maps of the smallest resolutions were taken from the last/deepest convolution layer, these feature maps are denoted as $F\in R^ {H\times W \times C}$. The feature maps contain high-level semantic information; however, the use of these coarse features may produce less accurate results, especially when dealing with images with complex structures, which could be due to factors such as the presence of disease.

**Figure 3:**
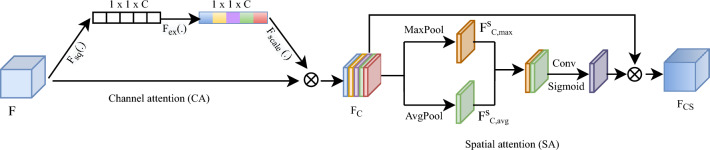
Architecture of the dual lesion attention (DLA) module that consists of two main submodules: channel attention (CA) and spatial attention (SA). Given the input feature map F, the CA module computes the corresponding channel-wise attention map $(F_c)$ and the SA module computes the final spatial-wise attention map $(F_{cs})$. $F_{sq} (.):$ squeeze operation, $F_{ex} (.):$ excitation operation, $F_{scale} (.):$ scaling operation.

*Feature refinement* To concentrate on the infection regions and refine the features for precise classification of pneumoconiosis into distinct categories, a dual attention mechanism was introduced, which sequentially integrated the CA and SA modules. This approach facilitates the extraction of discriminative features for accurate classification. The detailed structure of the proposed DLA model is illustrated in Fig. 3. This module takes feature maps F as input and uses the CA module to highlight the inter-channel relationship and the SA module to highlight inter-spatial relationship of the features related to lesions.

*Channel attention* In this study, we used a Squeeze and Excitation (SE) block^25^ to obtain channel-wise attention information. Each channel of a feature map represents a special feature detector. The CA module enables the recalibration of feature responses at the channel level by explicitly modeling the inter-dependencies between channels. For an input feature map $F\in R^ {H\times W \times C}$, first global average pooling is computed to generate a channel descriptor $F_{avg}^c \in R^ {1\times 1 \times C}$, which denotes average-pooled features. This process is known as the squeeze operation $(F_{sq})$. Then the descriptor is passed through a multi-layer perceptron (MLP) analyser, which consists of one hidden layer, to obtain the final channel-wise attention map $A_c \in R^ {1\times 1 \times C}$, a process known as the excitation operation $(F_{ex})$. To reduce parameter overhead, the size of the hidden layer in the MLP is set to C/r, where r represents the compression ratio. The channel-wise attention map is computed as

1 $$\begin{aligned} A_{c} = & Sig\left( {MLP\left( {AvgPool\left( F \right)} \right)} \right) \\   = & Sig\left( {W_{1} \left( {W_{0} \left( {F_{{avg}}^{c} } \right)} \right)} \right) \\ \end{aligned}$$

Where Sig represents the sigmoid activation function to normalise channel attention weights. MLP and AvgPool denote the operation of MLP and average pooling respectively. $W_0 \in R ^{(C/r) \times C}$ and $W_1 \in R ^{C \times (C/r) }$ are the weights of the MLP. The channel refined feature map is obtained by multiplying channel attention weights $A_c$ with the original feature map F. This operation is known as the scaling operation $(F_{scale})$, which can be defined as follows,

2 $$\begin{aligned} F_c\in A_c \otimes F \hspace{30em} \end{aligned}$$

where $\otimes$ represents element-wise multiplication, and the attention weights Ac are broadcast along spatial dimensions.

*Spatial attention* Not all locations within the feature maps contribute equally to a specific task. The SA module identifies the critical locations within the feature maps that require the network’s attention for processing. Inspired by the SA block proposed in CBAM^26^, an SA module is devised, which is then combined with the CA module for enhanced performance. The architecture of the SA module is shown in Fig. 3. To focus on disease-specific information, the SA mechanism is applied to the channel refined feature map $F_c\in R^ {H\times W \times C}$. First, average pooling and max pooling operations are employed to consolidate the channel information within the feature map, resulting in the generation of two 2D spatial feature descriptors: $F_{c,avg}^s \in R^ {H\times W \times 1}$ and $F_{c,max}^s \in R^ {H\times W \times 1}$, which denote averaged-pooled features and max-pooled features respectively. Subsequently, these two feature maps are concatenated, and a 7 x 7 convolution operation is applied to produce a 2D spatial attention map. In short, the computation of the spatial attention map can be described by:

3 $$A_{s} = Sig\left( {Conv^{{7 \times 7}} \left( {\left[ {F_{{c,avg}}^{s} ,F_{{c,max}}^{s} } \right]} \right)} \right)$$

where Sig represents the sigmoid activation function to normalise spatial attention weights. $Conv^{7\times 7}$ stands for the convolution operation with filter size 7 x 7, and [,] denotes the concatenation operation. The spatial refined feature map $F_{cs}\in R^ {H\times W \times C}$ is obtained by multiplying spatial attention weights $A_s$ with the channel refined feature map $F_c$.

4 $$\begin{aligned} F_{cs} = A_s \otimes F_c \end{aligned}$$

where $\otimes$ denotes element-wise multiplication, and the attention weights $A_s$ are broadcast across the channel dimensions during the multiplication process. By employing the DLA module, the focus is on disease-related features while simultaneously suppressing disease-irrelevant features.

*Classification* From the output layer of the backbone model, coarse features (F) are forwarded to the DLA module to emphasise the infection regions within the lungs, leading to the generation of refined features $F_{cs}$. The attentive features $F_{cs}$ are then fed into a fully connected layer followed by a SoftMax layer, to classify the input CXR images into different classes. Six different models were used, one each to classify the six zones. Each zone was assigned a category label of 0, 1, 2 or 3. To determine the overall classification label for the entire image, the predicted zone labels were combined as follows: the highest category among the six zones determines the ILO category for the entire image. For instance, if the predicted categories for the six zones of an image are [RUZ: 1, RMZ: 0, RLZ: 0, LUZ: 2, LMZ: 1, LLZ: 0], the image would be classified as ILO category 2. For all models, a similar procedure was applied to calculate image-level classification from zone-level classification.

*Ethics approval and consent to participate* CSIRO Health and Medical Human Research Ethics Committee, Australia, granted approval for this research (approval number: LR 22/2016), and waived a requirement of informed consent since data were evaluated retrospectively and pseudonymously, and was solely obtained for treatment purposes. All methods were performed in accordance with the relevant institutional guidelines and regulations, and all data used for the research were de-identified.

**Table 1:** Details of the datasets used in the experiments for pneumoconiosis classification.

Database name	ILO category				Total images
	Cat-0	Cat-1	Cat-2	Cat-3	
GMH	13	98	64	47	222
MIC	64	15	7	1	87
RWSH	0	325	15	3	343
CSH	503	0	0	0	503
SVH	35	0	0	0	35
NIOSH	8	31	14	9	62
Total images	623	469	100	60	1252

## Experimental setup

*Data description* To develop a pneumoconiosis classification model, CXR images from patients of different ILO categories were collected by collaboration with different organisations, including Good Morning Hospital (GMH) in South Korea, a Medical Imaging Clinic (MIC), an independent regulator of worker safety and health (RWSH), Coal Services Health (CSH), St Vincent’s Hospital (SVH) and The National Institute for Occupational Safety and Health (NIOSH), as shown in Table 1. Among the 1,252 CXR images, only 62 were obtained from the publicly available NIOSH dataset. Since the images were acquired from diverse sources, a histogram matching algorithm^29^ was employed to adjust the histograms of all the images uniformly. Ground truth of four pneumoconiosis categories based on ILO guidelines was provided by radiologists from Good Morning Hospital and Lung Screen Australia.

*System implementation* The model was implemented using Keras with TensorFlow as the backend. Experiments were conducted on a Dell C4140 server within a High-Performance Computing (HPC) cluster, equipped with 2 x Intel Xeon 6130 CPUs (16C, 2.1GHz, 125W), 192GB RAM (12 x 16GB) and 4 x Nvidia V100 GPUs (32GB NVLink) for faster computation. The Adam optimiser was utilised for all models with an initial learning rate set to 0.0001, a weight decay factor of 0.2 and a patience value of 10. The batch size was configured as 4 and the number of epochs was set to 100. To prevent overfitting and save time, early stopping based on validation loss was employed. All the images from the six zones were resized before training and testing the model. To maintain the original aspect ratio, the average height and width of all zone images were first measured. Then, the image height was set to 256 and the width calculated using the corresponding aspect ratio. For instance in the RUZ zone, the aspect ratio for height and width was (1:1.25). Therefore, for an image height of 256, the image width was set to 320. Five-fold cross validation was used to evaluate the performance of the classification models and samples from each class were equally distributed among different folds. We calculate the standard deviation as percentages (%).

*Evaluation metrics* The classification performance of the models was assessed using accuracy (ACC), sensitivity (SEN), specificity (SPE), F1 score (F1), and area under receive operation curve (AUC), which are defined as follows:

5 $$\begin{aligned} ACC= & {} \frac{(TN + TP)}{(TN + TP + FN + FP) } \hspace{20em} \end{aligned}$$

6 $$\begin{aligned} SEN= & {} \frac{TP}{(TP + FN)} \hspace{25em} \end{aligned}$$

7 $$\begin{aligned} SPE= & {} \frac{TN}{(TN + FP)} \hspace{25em} \end{aligned}$$

8 $$\begin{aligned} F1= & {} \frac{2TP}{(2TP + FP + FN)} \hspace{22em} \end{aligned}$$

**Table 2:** Comparison of classification performance of different models at zone level.

Zone	DenseNet121			Xception			EfficientB4			Proposed		
	Acc	F1	AUC	Acc	F1	AUC	Acc	F1	AUC	Acc	F1	AUC
LLZ	0.826 ± 0.96	0.738 ± 0.66	**0.847 ± 1.40**	0.821 ± 1.20	**0.744 ± 1.49**	0.843 ± 1.92	0.826 ± 1.16	0.735 ± 0.83	0.851 ± 1.37	**0.827 ± 1.11**	0.739 ± 1.66	0.844 ± 2.44
LMZ	**0.867 ± 2.11**	0.773 ± 3.51	0.893 ± 1.91	**0.867 ± 1.95**	0.777 ± 2.62	0.879 ± 2.67	0.858 ± 1.76	0.772 ± 2.59	0.875 ± 1.78	**0.867 ± 1.59**	**0.780 ± 2.07**	**0.896 ± 2.12**
LUZ	0.888 ± 1.22	0.819 ± 2.16	**0.914 ± 1.08**	0.885 ± 1.71	0.811 ± 2.87	0.901 ± 2.09	**0.891 ± 1.98**	**0.820 ± 2.82**	0.909 ± 1.32	0.889 ± 1.51	0.816 ± 2.17	0.898 ± 1.81
RLZ	0.824 ± 2.64	0.727 ± 3.17	0.868 ± 2.79	0.825 ± 2.72	0.733 ± 3.43	0.873 ± 2.41	0.818 ± 1.03	0.728 ± 1.69	0.862 ± 1.70	**0.841 ± 1.83**	**0.740 ± 2.90**	**0.885 ± 1.92**
RMZ	0.844 ± 2.24	0.743 ± 2.51	0.887 ± 1.91	0.841 ± 1.50	0.754 ± 3.15	0.893 ± 2.17	0.853 ± 1.03	0.762 ± 1.97	**0.901 ± 0.93**	**0.856 ± 0.82**	**0.777 ± 2.29**	**0.901 ± 1.66**
RUZ	0.885 ± 0.87	0.807 ± 2.15	0.911 ± 1.04	**0.891 ± 0.99**	0.806 ± 1.27	0.908 ± 0.98	0.888 ± 0.91	**0.816 ± 1.56**	0.912 ± 2.16	0.888 ± 0.75	0.811 ± 1.48	**0.914 ± 1.38**
Avg.	0.856 ± 1.67	0.768 ± 2.36	0.886 ± 1.68	0.8555 ± 1.67	0.771 ± 2.47	0.883 ± 2.04	0.856 ± 1.31	0.772 ± 1.91	0.885 ± 1.54	**0.861 ± 1.48**	**0.777 ± 2.07**	**0.890 ± 1.53**

Best results indicated in bold.

**Table 3:** Classification performance comparison of different models at the image level.

Models	Multi-class classification				
	ACC	SEN	SPE	F1	AUC
DenseNet121	0.8258 ± 1.88	0.7188 ± 1.46	0.8766 ± 1.98	0.7260 ± 1.69	0.7977 ± 1.60
ResNet50	0.8248 ± 2.40	0.7229 ± 2.81	0.8689 ± 2.71	0.7293 ± 3.06	0.7959 ± 2.63
Inceptionv3	0.8288 ± 0.77	0.7204 ± 1.20	0.8762 ± 1.12	0.7278 ± 1.31	0.7983 ± 1.06
Xception	0.8279 ± 2.55	0.7189 ± 3.04	0.8838 ± 1.93	0.7294 ± 2.92	0.8013 ± 2.23
EfficientNetB4	0.8323 ± 1.01	0.7293 ± 1.23	0.8860 ± 1.00	0.7394 ± 0.92	0.8076 ± 0.71
DLA-Net (Proposed)	**0.8562 ± 1.61**	**0.7643 ± 1.44**	**0.8941 ± 1.81**	**0.7687 ± 1.53**	**0.8292 ± 1.53**

Best results are indicated in bold.

where TP, TN, FP and FN represent the number of true positives, true negatives, false positives and false negatives, respectively. TP, TN, FP and FN were calculated from the confusion matrix. The area under the curve (AUC) was calculated by analysing all possible combinations of true positive rate and false positive rate through threshold adjustments on the prediction results. The AUC reflects the probability that a classification model ranks a randomly chosen positive instance higher than a randomly chosen negative case. The F1 score is the harmonic mean of true positives and sensitivity. AUC and F1-score are the most commonly used metrics to evaluate the overall performance of a classification model. SEN and SPE indicate the proportions of correctly identified positive and negative samples, respectively. Accuracy indicates the percentage of correctly classified samples, considering both positive and negative samples. Since the dataset was imbalanced, the metrics were computed using a weighted average approach, taking into account the number of actual occurrences of each class in the dataset.

## Results

This section initially presents the results of zone-level classification and subsequently covers image-level classification results.

### Zone-level classification

The classification results obtained from four network architectures for the six zones are presented in Table 2. The average performance across all zones indicates that the proposed model outperforms the other network architectures in terms of all metrics. There is a notable disparity in performance between the upper and lower zones using the same model. At the zone level, the top zones of both lungs (LUZ and RUZ) demonstrate outstanding performance in classification, while lower performance is observed in the bottom two zones (LLZ and RLZ). This discrepancy may be attributed to the presence of the hilum, which is situated approximately midway down each lung. Moving from the hilum towards the periphery in the lower zones, there is a gradual reduction in anatomical lung markings, which can affect the performance of classification. In contrast, the top left and right zones exhibit higher performance due to more easily identifiable radiographic abnormalities associated with pneumoconiosis in those regions.

The findings also demonstrate that the proposed network outperforms all other network architectures, particularly in the middle two zones (LUZ, RUZ) and RLZ. This could be attributed to the significant presence of opacities in the middle zone, enabling the proposed network to extract discriminative features from diseased lung regions effectively and leading to superior performance. However, in LUZ, EfficientB4 outperforms all other networks in terms of Acc and F1 evaluation metrics, while for other zones the performance of different network architectures is comparable.

### Image-level classification

A comparative analysis of image-level classification performance of the proposed model against several state-of-the-art methods, including DenseNet121^22^, ResNet50^21^, Inceptionv3^19^, Xception^20^ and EfficientNetB4^30^ was conducted, as shown in Table 3. To ensure fair comparison, all models were trained and evaluated on the same dataset using similar parameter settings. The results clearly demonstrate that the proposed model outperforms the other state-of-the-art methods by a significant margin across all evaluation metrics. When compared to the EfficientNetB4^30^ model, which achieved the second-best performance, the proposed model exhibits substantial improvement, with an increase in accuracy, sensitivity, specificity, F1 score and AUC of 2.39%, 3.0%, 0.81%, 2.93% and 2.16%, respectively. Similarly, when compared to the baseline Xception^20^ model, the addition of the DLA module enhances the accuracy, sensitivity, specificity, F1 score and AUC by 2.83%, 4.54%, 1.03%, 3.93% and 2.79% respectively.

**Table 4:** Comparison of image-level detection performance across different models.

Models	Binary classification				
	ACC	SEN	SPE	F1	AUC
DenseNet121	0.8562 ± 2.75	0.8562 ± 2.75	0.8558 ± 2.77	0.8555 ± 2.85	0.7958 ± 1.57
ResNet50	0.8459 ± 2.34	0.8459 ± 2.34	0.8455 ± 2.32	0.8457 ± 2.34	0.7947 ± 2.95
Inceptionv3	0.8538 ± 1.04	0.8538 ± 1.04	0.8535 ± 1.02	0.8536 ± 1.03	0.7981 ± 0.91
Xception	0.8563 ± 3.43	0.8563 ± 3.43	0.8557 ± 3.46	0.8551 ± 3.54	0.8013 ± 2.51
EfficientNetB4	0.8570 ± 1.16	0.8570 ± 1.16	0.8564 ± 1.16	0.8561 ± 1.21	0.8071 ± 1.18
DLA-Net (Proposed)	**0.8841 ± 2.22**	**0.8841 ± 2.22**	**0.8840 ± 2.22**	**0.8841 ± 2.22**	**0.8291 ± 2.29**

The best results are highlighted in bold.

In the zone-level experiments, the average performances of the proposed method is marginally better than other methods. However, at the image level, the proposed method significantly outperforms others. The primary reason is that to determine the overall classification label for a CXR image, the predicted zone labels are combined as follows: the highest category among the six zones determines the ILO category for the CXR image. Therefore, for correct image-level classification, all six zones should be classified correctly. If five zones are classified correctly but only one zone is classified incorrectly, then the overall image classification may be incorrect. This is because if the category of incorrectly classified zone is the largest among all zones, the category will be used as the category of the overall image-level classification. Hence, even in the zone-level classification, the average performance of the proposed method is slightly better than others, this has made a big difference in image-level classification, leading to the significant improvement of the performance of the proposed model.

**Figure 4:**
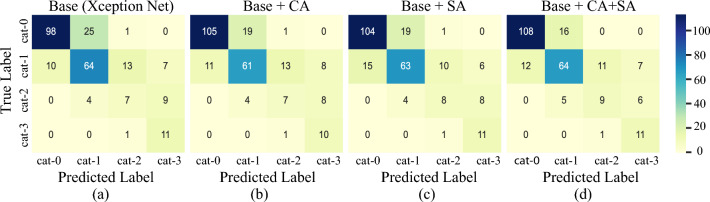
Confusion matrices for pneumoconiosis classification for four different models. (**a**) Base (Xception Net), (**b**) Base +CA, (**c**) BASE+SA, and (**d**) Base+CA+SA (the proposed model).

To assess the effectiveness of the proposed model, we also conducted a comparison of various models for pneumoconiosis detection or binary class classification, as presented in Table 4. Similar to multi-class classification, the proposed model outperforms all other models for pneumoconiosis detection. EfficientNetB4^30^ achieved the second-highest results, while ResNet50^21^ showed the lowest results. These results demonstrate that the DLA module plays a crucial role in enabling the model to focus on the lesion areas inside the lungs, resulting in improved performance and better identification of pneumoconiosis-related abnormalities.

To gain a better understanding of the effectiveness of the attention module, we used the confusion matrices to visualise the classification results, as depicted in Fig. 4. The confusion matrices reveal that all methods tend to misclassify images with the adjacent severity classes, which is consistent with the clinical classification rules. Across all models, the recognition performance for cat-3 is generally high, while the performance for cat-2 is relatively low compared to the other categories. For instance, as shown in Fig. 4a, the base model correctly identifies only 7 out of 20 cat-2 images, misclassifying 4 images as cat-1 and 9 images as cat-3. There are two possible reasons for the misclassification of cat-2: (1) The model was trained on only a limited number of cat-2 images. To address the data imbalance issue, data augmentation techniques were applied, however, the biased classification results indicate that this technique was not effective. (2) Images with cat-2 visually resemble cat-1 and cat-3 images (see Fig. 1), making the classification challenging for the network to learn the subtle differences between cat-1 and cat-2 as well as between cat-2 and cat-3, resulting in classification errors.

However, after incorporating the CA or SA module into the baseline model, the classification performance is improved, as depicted in Figs. 4b,c. Performance is further improved when the DLA module is employed, as shown in Fig. 4d. For example, the proposed model correctly identifies 9 out of 20 cat-2 images, misclassifying 5 images as cat-1 and 6 images as cat-3. These results indicate that the proposed model is capable of learning more subtle differences between different categories by focussing on the diseased regions inside the lungs. On the other hand, all models demonstrate higher accuracy in classifying images with cat-3 compared to other categories, despite being trained on only a limited number of cat-3 images. For example, both the base model and the proposed model correctly identify 11 out of 12 cat-3 images, with only 1 image being identified as cat-2. This may be attributed to the relatively obvious opacities presented in cat-3 images and the significant visual differences between cat-3 images and images of other categories (see Fig. 1). The presence of discriminative opacity features in cat-3 images makes them easier to identify.

### Feature visualisation using t-SNE

**Figure 5:**
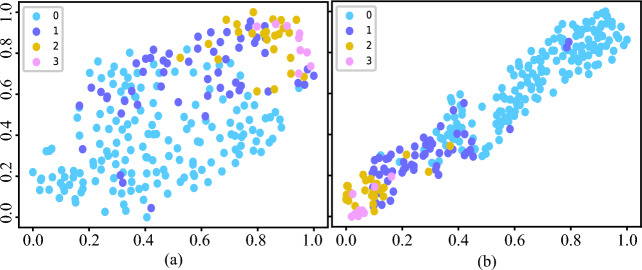
t-SNE visualisation plot of feature distribution for four different types of pneumoconiosis images generated by (a) Xception Net and (b) DLA-Net (proposed).

The classification results show that the proposed DLA-Net can extract more comprehensive and discriminative features, leading to improved classification performance. To support this assertion, a t-distributed Stochastic Neighbor Embedding (t-SNE) analysis^31^ was conducted to visualise feature vectors generated by the Xception network and the proposed networks. The t-SNE algorithm is a dimensionality reduction technique that projects high-dimensional data into a two-dimensional space^31^. The high dimensional data in this context are features extracted from the last layer of a trained pneumoconiosis classification model. For feature extraction, the middle zone (RMZ) was chosen due to its higher density of opacities. Two network architectures were considered: Xception and the proposed DLA-Net. The resulting two-dimensional t-SNE embedding plots using features from both network architectures are presented in Figs. 5a,b respectively. In the figures, points represented by four distinct colors correspond to four ILO categories. It is observed that in comparison to DLA-Net, the features generated by the Xception network for categories cat-0, cat-1 and cat-2 exhibit more overlap. Through t-SNE visualisation, it is suggested that DLA-Net extracts more discriminative features than Xception Net, thereby contributing to an enhancement in classification performance. The t-SNE visualisation also shows consistency with the results shown in Fig. 4.

### **Interpretability using saliency map**

**Figure 6:**
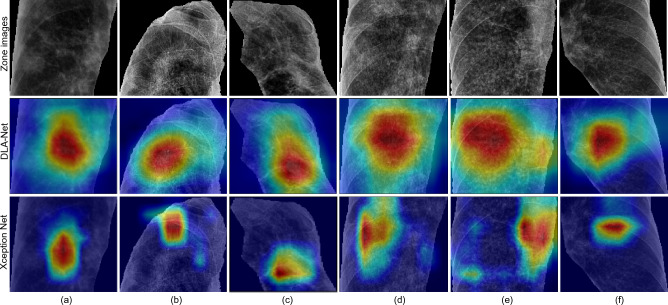
Grad-CAM visualisation results for six zone images (a-f). The top row shows the original zone images, while the middle and bottom rows show the saliency maps generated by DLA-Net and Xception Net, respectively.

To visualise the areas of the greatest concern identified by the last layer of Xception Net and the proposed DLA-Net, Grad-CAM was applied, as shown in Fig. 6. The figure shows 6 zone images from the test set. The results suggest that Xception Net struggles to focus on the key object, i.e., opacities inside the zone. In contrast, the proposed DLA-Net (Xception Net + DLA module) uses the CA module to focus on the most important channels in the feature maps and the SA module to highlight the spatial details of the infected regions. For example in Fig. 6a, RMZ contains opacities at the left and bottom part of the zone that are not highlighted by Xception Net. Similarly in Fig. 6b, the main lesions are not properly highlighted by Xception Net. However, the proposed model can highlight the lesions more accurately compared to Xception Net. As a result, the proposed model can extract discriminative features from infected regions by paying better attention. This discriminative feature extraction helps the model to improve classification performance on pneumoconiosis.

### Ablation study

In this section, we explore the effectiveness of the backbone architecture, attention modules and compression ratio.

*Backbone architecture selection* The DLA module can be seamlessly incorporated into various backbone architectures to enhance their performance. To assess the effectiveness of different backbone architectures, we used several pre-trained models, including DenseNet121^22^, ResNet50^21^, Inceptionv3^19^, EfficientNetB4^30^ and Xception^20^. The performance of these models, both with and without the DLA modules, is presented in Table 5. To ensure fair comparison, consistent parameter settings were maintained such as batch size, optimiser, number of epochs and data augmentation techniques during the training of all models. The results indicate that the DLA module positively impacts the performance of all the networks, leading to improved classification results. Among the tested architectures, the model with Xception^20^ as the backbone achieved the best performance. While the performances of Xception^20^ and EfficientNetB4^30^ architectures were comparable, Xception^20^ exhibited slightly superior results. Therefore, considering overall performance, Xception^20^ was selected as the backbone architecture for the proposed model.

**Table 5:** Comparison of the performance of different backbone architectures.

Models	Multi-class classification				
	ACC	SEN	SPE	F1	AUC
DenseNet121	0.8258 ± 1.88	0.7188 ± 1.46	0.8766 ± 1.98	0.7260 ± 1.69	0.7977 ± 1.60
DLA-DenseNet121	0.8458 ± 1.97	0.7525 ± 2.80	0.8858 ± 1.65	0.7575 ± 2.73	0.8191 ± 2.22
ResNet50	0.8248 ± 2.40	0.7229 ± 2.81	0.8689 ± 2.71	0.7293 ± 3.06	0.7959 ± 2.63
DLA-ResNet50	0.8348 ± 1.25	0.7388 ± 1.79	0.8738 ± 2.05	0.7415 ± 2.36	0.8063 ± 1.85
Inceptionv3	0.8288 ± 0.77	0.7204 ± 1.20	0.8762 ± 1.12	0.7278 ± 1.31	0.7983 ± 1.06
DLA-Inceptionv3	0.8302 ± 1.04	0.7188 ± 1.53	0.8807 ± 0.59	0.7269 ± 1.44	0.7998 ± 0.89
EfficientNetB4	0.8323 ± 1.01	0.7293 ± 1.23	0.8860 ± 1.00	0.7394 ± 0.92	0.8076 ± 0.71
DLA-EfficientNetB4	0.8489 ± 1.62	0.7468 ± 2.91	**0.8966 ± 1.07**	0.7550 ± 2.41	0.8217 ± 1.75
Xception	0.8279 ± 2.55	0.7189 ± 3.04	0.8838 ± 1.93	0.7294 ± 2.92	0.8013 ± 2.23
DLA-Xception	**0.8562 ± 1.61**	**0.7643 ± 1.44**	0.8941 ± 1.81	**0.7687 ± 1.53**	**0.8292 ± 1.53**

Best results indicated in bold.

*Impact of attention modules* To assess the effectiveness of the proposed attention modules, ablation studies were conducted where the individual contributions of the CA module, SA module as well as their combination in parallel and sequential configurations (proposed model) were examined. The results of these ablation studies are presented in Table 6.

**Table 6:** Effect of the attention modules on the model performance.

Method	Multi-class classification				
	ACC	SEN	SPE	F1	AUC
Base	0.8279 ± 2.55	0.7189 ± 3.04	0.8838 ± 1.93	0.7294 ± 2.92	0.8013 ± 2.23
Base + CA	0.8425 ± 0.54	0.7356 ± 1.10	0.8900 ± 0.85	0.7447 ± 0.93	0.8128 ± 0.69
Base + SA	0.8372 ± 1.62	0.7404 ± 2.06	0.8779 ± 1.88	0.7447 ± 2.24	0.8091 ± 1.88
Base + CA + SA (Parallelly)	0.8520 ± 2.23	0.7556 ± 3.10	0.8934 ± 1.54	0.7616 ± 2.86	0.8245 ± 2.23
Base + CA + SA (Sequentially)	**0.8562 ± 1.61**	**0.7643 ± 1.44**	**0.8941 ± 1.81**	**0.7687 ± 1.53**	**0.8292 ± 1.53**

Best results indicated in bold.

From the observations in Rows 2 and 3 of Table 6, it is evident that both the CA and SA modules significantly improve the classification performance, and their performances are comparable to each other. Furthermore, the combination of both attention modules simultaneously leads to further improvement in the classification performance. This can be attributed to the fact that the dual attention modules allow the model to focus more on the affected regions and extract discriminative features that are effective for pneumoconiosis classification.

However, it is worth noting that the model achieves slightly better performance when the CA and SA modules are combined sequentially (Row 5 in Table 6) compared to when they are combined in parallel (Row 4 in Table 6). This sequential combination allows for sequential integration of the CA and SA modules, enabling the refinement of features and enhancing the ability of the model to capture relevant information. Overall, these ablation studies highlight the significant contributions of both the CA and SA modules, and the sequential combination of these modules further improves the performance of the proposed model in pneumoconiosis classification.

**Table 7:** Effect of the compression ratio on the model performance. Best results indicated in bold.

Reduction ratio r	Multi-class classification				
	ACC	SEN	SPE	F1	AUC
4	0.8444 ± 1.42	0.7380 ± 1.93	0.8949 ± 1.74	0.7475 ± 1.96	0.8164 ± 1.68
8	0.8419 ± 1.70	0.7492 ± 2.24	0.8855 ± 1.73	0.7558 ± 2.26	0.8173 ± 1.81
16	**0.8562 ± 1.61**	**0.7643 ± 1.44**	0.8941 ± 1.81	**0.7687 ± 1.53**	**0.8292 ± 1.53**
32	0.8557 ± 1.98	0.7532 ± 2.80	**0.8989 ± 1.83**	0.7602 ± 3.04	0.8260 ± 2.22

*Impact of compression ratio r* In this section, the impact of different compression ratios (r) on the classification performance of the model is evaluated. The evaluation results are summarised in Table 7. As observed from the table, the classification performance of the proposed model shows an increasing trend as the value of r increases from 2 to 16. However, beyond a certain point, when the value of r continues to increase, the performance starts to decrease. This behaviour may be attributed to the following factors.

For smaller values of the compression rate, the MLP module contains an excessive amount of redundant information. This redundancy leads to a decrease in the classification performance as the model becomes overwhelmed with abundant data. On the other hand, for larger values of the compression rate, the MLP module may miss out on crucial features necessary for accurate classification. This deficiency in capturing essential information results in misclassification and a subsequent decrease in performance. Based on these observations, a compression rate of 16 is selected for this study, as it results in the best classification performance. This value strikes a balance between preserving relevant information and avoiding excessive redundancy in the MLP module.

## Limitations and future work

While the proposed DLA-Net has shown superior performance compared to other state-of-the-art methods for pneumoconiosis classification, there are some limitations in this study. First, the models were trained and evaluated using an imbalanced dataset. The datasets, particularly the GMH dataset, were collected from patients with an average age of over 70 years. Therefore, the presence of coexisting diseases for some patients makes it challenging for the model to differentiate between images of different categories. Moreover, some images in the GMH dataset contain artifacts that can also impact the performance of the model. Secondly, although the proposed method achieved good results compared to other methods, there is room for improvement, especially in accurately identifying cat-2 images. Further research is needed to enhance model performance in this regard. Thirdly, it is important to note that the models may not generalise to differentiate pneumoconiosis from other lung diseases with similar pathology, such as COVID-19.

In future studies, these limitations will be addressed to further improve the classification performance. One approach would be to collect more annotated images, particularly for cat-2 and cat-3, to address the limited and imbalanced dataset issue. An alternative solution could involve generating synthetic images using Generative Adversarial Networks (GANs) or other deep learning-based models. Additionally, model performance may be enhanced by integrating other clinical information such as age, working history and lung function. Furthermore, incorporating more annotated CXR images from patients with various lung diseases may contribute to better model performance and generalisability.

Recently, transformers have found successful applications in medical image analysis, including segmentation and classification. In certain scenarios, transformer-based networks have outperformed CNN-based methods^32^. In the future, we plan to incorporate this attention-based module in a transformer-based model pre-trained on the ImageNet dataset.

In conclusion, while the proposed DLA-Net has demonstrated promising results, there are several avenues for future research to overcome the limitations and further enhance the performance of the proposed model for accurate classification of pneumoconiosis.

## Conclusion

In the field of medical image analysis, CNN-based methods have demonstrated impressive performance. However, they face challenges when it comes to accurately classifying pneumoconiosis, particularly in CXR images with complex structures. To address this, a CNN-based method called DLA-Net is proposed for pneumoconiosis classification. The proposed model incorporates dual attention mechanisms, which allow for the extraction of powerful and discriminative features by focussing on the lung regions that exhibit lesions. This attention mechanism enhances the model’s ability to represent features effectively. DLA-Net was trained on specific subregions of the lungs to predict the opacity level of each zone individually. Subsequently, each CXR image was classified based on the predictions obtained from these subregion-based predictions. All the evaluated models showed better performance for the upper lung zones compared to the lower lung zones. Extensive experiments validated the effectiveness of the proposed model, which outperformed state-of-the-art models for pneumoconiosis classification.

## Data Availability

Private datasets are not publicly available due to restrictions in data sharing agreements with third party X-ray providers. These providers include Good Morning Hospital (GMH), South Korea, Coal Services Health (CSH), Australia, and St Vincent’s Hospital (SVH), Sydney. The public dataset NIOSH is available at https://www.cdc.gov/niosh/learning/b-reader/start/1.html.
